# New Multidrug-Resistant *Salmonella enterica* Serovar Anatum Clone, Taiwan, 2015–2017

**DOI:** 10.3201/eid2501.181103

**Published:** 2019-01

**Authors:** Chien-Shun Chiou, Yu-Ping Hong, Ying-Shu Liao, You-Wun Wang, Yueh-Hua Tu, Bo-Han Chen, Yi-Syong Chen

**Affiliations:** Centers for Disease Control, Taichung, Taiwan

**Keywords:** *Salmonella enterica* serovar Anatum, antimicrobial resistance, multidrug resistance, molecular epidemiology, bacteria, Taiwan

## Abstract

In 2011, a *Salmonella enterica* serovar Anatum clone emerged in Taiwan. During 2016–2017, infections increased dramatically, strongly associated with emergence and spread of multidrug-resistant strains with a plasmid carrying 11 resistance genes, including *bla*_DHA-1_. Because these resistant strains infect humans and food animals, control measures are urgently needed.

*Salmonella*, a prevalent foodborne pathogen that causes zoonoses worldwide, comprises 2 species, *Salmonella enterica* and *S*. *bongori*, and ≈2,600 serovars (*1*). In Taiwan, salmonellosis has been primarily caused by the *S. enterica* serovars Enteritidis, Typhimurium, Stanley, Newport, and Albany, which together caused 70% of salmonellosis infections during 2004–2012 (*2*). During this period, *Salmonella* Anatum was not prevalent, causing only 0.4% of the infections. However, since 2015, *Salmonella* Anatum infections have increased, and most isolates are multidrug resistant (MDR). We report the epidemiologic trend of *Salmonella* Anatum infection of humans, the clonal relationships among strains recovered during 2004–2017, and the resistance mechanism of the newly emerging MDR strains.

## The Study

To investigate the epidemiologic trend, we analyzed the data in the *Salmonella* fingerprint database constructed by the Taiwan Centers for Disease Control. The database comprises demographic and experimental data, including pulsed-field gel electrophoresis (PFGE) fingerprints obtained by using the PulseNet standardized PFGE protocol (*3*), serotypes obtained using PFGE pattern comparison and conventional methods (*4*), and antimicrobial drug susceptibility testing results for isolates collected from hospitals nationwide. We conducted whole-genome sequencing for 68 *Salmonella* Anatum isolates from humans and animals and 9 isolates from chicken carcasses and abbatoir environments by using the Illumina MiSeq platform (https://www.illumina.com) and identified resistance genes, incompatibility groups of plasmids, and sequence types by using the whole-genome sequencing data. To investigate clonal relationships and locations of resistance genes, we constructed a dendrogram for *Salmonella* Anatum strains with whole-genome single-nucleotide polymorphism profiles to assess genetic relatedness among strains and determined the complete genomic sequence of *Salmonella* Anatum strain R16.0676 with whole-genome sequencing data generated by using a MinION nanopore sequencer (https://nanoporetech.com/products/minion) and an Illumina MiSeq sequencer. To investigate mobility of resistance plasmids, we conducted conjugation experiments to transfer the resistance genes–carrying (R) plasmid from *Salmonella* Anatum strain R16.0676 into recipient *Escherichia coli* C600 and transferred an R plasmid from an *E. coli* transconjugant back to a rifampin-resistant mutant of *Salmonella* Anatum strain R13.0957 (Appendix).

**Figure 1:**
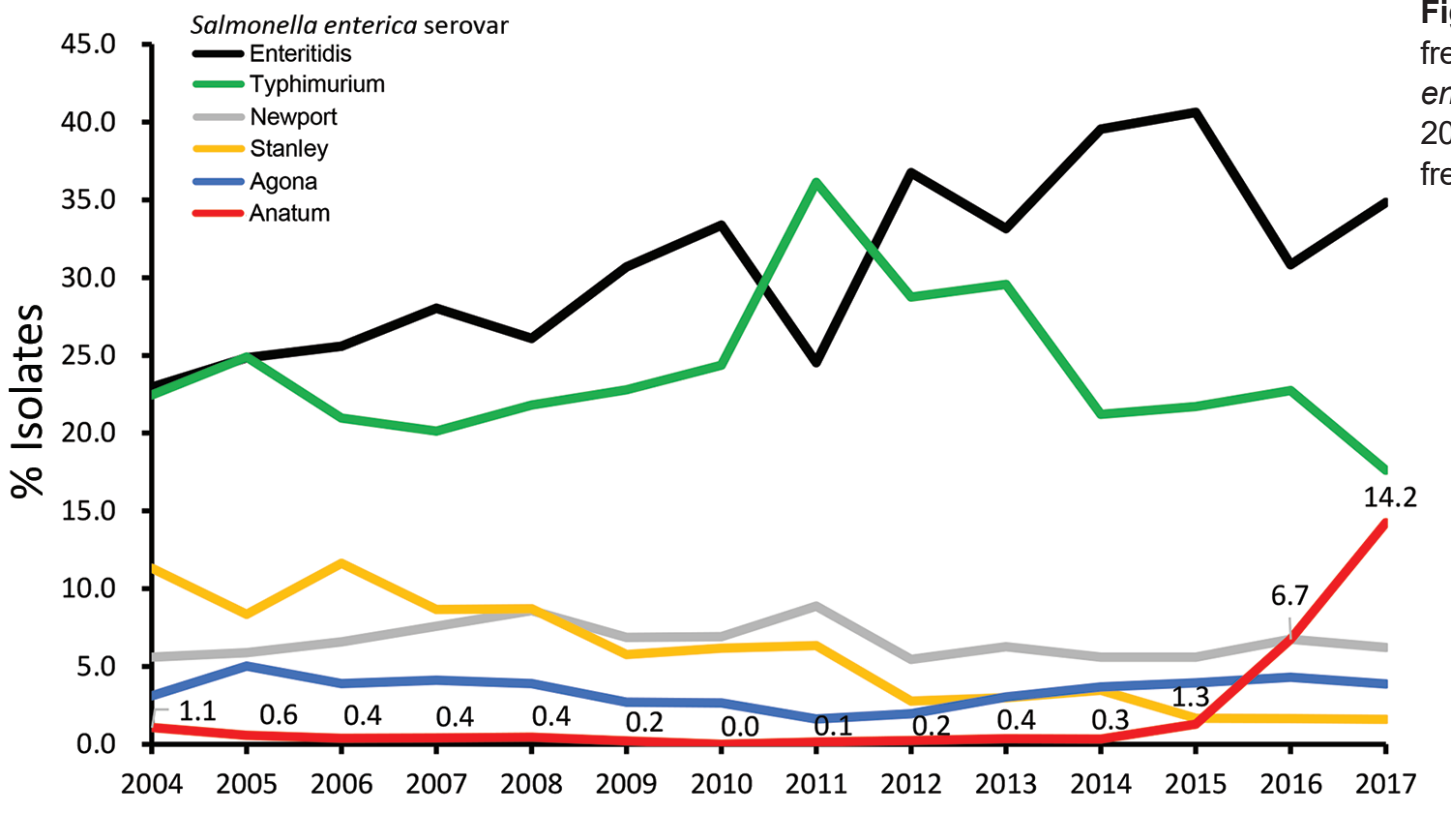
Distribution of the 6 most frequently identified *Salmonella* *enterica* serovars in Taiwan, 2004–2017. Numbers indicate increasing frequency of *Salmonella* Anatum.

The *Salmonella* fingerprint database of the Taiwan Centers for Disease Control contained PFGE fingerprints for 34,160 *Salmonella* isolates recovered during 2004–2017, of which antimicrobial drug sensitivity test results were available for 23,018. *Salmonella* Anatum was not a prevalent serovar among those collected during 2004–2014 (Figure 1). However, the number of *Salmonella* Anatum infections increased in 2015 and subsequently underwent another sharp increase in 2016 and 2017. In 2017, *Salmonella* Anatum accounted for 14.2% of *Salmonella* infections in Taiwan and ranked as the third most frequently identified serovar. 

**Figure 2:**
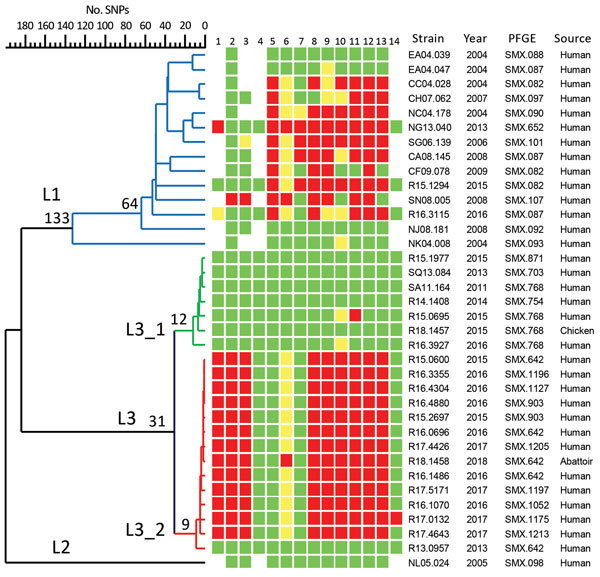
Dendrogram of 36 representative *Salmonella enterica* serovar Anatum strains from Taiwan, 2004–2017, constructed with whole-genome SNP profiles with 883 SNPs. The complete genomic sequence of *Salmonella* Anatum strain GT-38 (GenBank accession no. CP013226) was used as the reference for SNP calling. Red, resistant; yellow, intermediate; green, susceptible. Lanes: 1, cefoxitin; 2, cefotaxime; 3, ceftazidime; 4, ertapenem; 5, nalidixic acid; 6, ciprofloxacin; 7, gentamicin; 8, ampicillin; 9, chloramphenicol; 10, streptomycin; 11, sulfamethoxazole; 12, tetracycline; 13, sulfamethoxazole/trimethoprim; 14, colistin. L, lineage; PFGE, pulsed-field gel electrophoresis; SNP, single-nucleotide polymorphism.

Whole-genome single-nucleotide polymorphism analysis of *Salmonella* Anatum recovered from humans during 2004–2017 revealed 3 distinct lineages (Figure 2). Strains of lineage (L) 1 were either pansusceptible or MDR; they mostly appeared during 2004–2009 (Appendix Table 2). L2 comprised only 2 isolates, which emerged in 2005 and were pansusceptible. L3 comprised 2 sublineages; sublineage (SL) 3_1, first detected in 2011, was mostly pansusceptible, whereas SL3_2, which first emerged in 2013, was mostly MDR. The MDR strains of SL3_2 first appeared in 2015 and were resistant or of reduced susceptibility to 10 of the 14 antimicrobial drugs tested. SMX.642 was the predominant MDR strain, but the first 2 isolates recovered in 2013 were pansusceptible. Of the 9 isolates from chicken carcasses and abattoir environments, 5 belonged to SL3_1 and 4 to SL3_2. The new clone (L3) accounted for 91.9% of the total *Salmonella* Anatum infections during 2004–2017 and 99.6% in 2017. MDR strains accounted for 90.3% of the new clone recovered during 2011–2017 and 94.1% in 2017. All *Salmonella* Anatum isolates sequenced belonged to sequence type 64.

The chromosomal sequence of strain R16.0676 was 4,674,190 bp (GenBank accession no. CP029800) and was not noted to carry any horizontally transferable resistance gene. R16.0676 harbored 2 plasmids, which were designated pR16.0676_90k (90,137 bp; IncC; accession no. CP029802) and pR16.0676_34k (34,063 bp; IncN3; accession no. CP029801). pR16.0676_90k harbored 11 resistance genes, *aadA2*, *bla*_DHA-1_, *dfrA23*, *floR*, *lnu(F)*, *qnrB4*, *strA*, *strB*, *sul1*, *sul2*, and *tet(A)*, which were distributed in 2 antimicrobial resistance islands, ARI1 and ARI2 (Appendix Figure, panel A). ARI1 carried 5 resistance genes, *floR*, *strA*, *strB*, *sul2*, and *tet(A)*, and was found in many IncC plasmids in the National Center for Biotechnology Information database (*5*). ARI2 carried the other 6 resistance genes, *aadA2*, *bla*_DHA-1_, *dfrA23*, *lnu(F)*, *qnrB4*, and *sul1*. The resistance genes could confer resistance to cefoxitin, cefotaxime, ceftazidime, ampicillin, chloramphenicol, streptomycin, sulfonamide, tetracycline, and trimethoprim and reduced susceptibility to ciprofloxacin as shown by antimicrobial susceptibility testing (Figure 2). pR16.0676_90k shared 79% sequence identity with a 272-kb plasmid, pECAZ155_KPC (GenBank accession no. CP019001.1), which harbored only the sequence of ARI1 but not ARI2. pR16.0676_34k did not carry any resistance gene (Appendix Figure, panel B), but it shared 98% sequence identity with a 34.8-kb plasmid, pN-Cit (GenBank accession no. JQ996149.1). All MDR SL3_2 isolates, including the 4 isolates recovered from the abattoirs, harbored an IncC plasmid and the same 11 resistance genes identified in strain R16.0676. Strain R17.0132 acquired an additional *mcr-1* gene and was resistant to colistin (Figure 2). We did not obtain any transconjugants with pR16.0676_90k, but we did obtain a transconjugant with a composite plasmid, which had the same sequences as pR16.0676_90k and pR16.0676_34k (Appendix Figure, panel C). This 125-kb composite plasmid probably resulted from insertion of pR16.0676_90k into pR16.0676_34k through an insertion sequence 26–mediated transposition process. The resulting plasmid acquired an additional copy of insertion sequence 26 and an 8-bp tandem repeat in the insertion site. More than a dozen genes are typically required for conjugation (*6*). pR16.0676_90k harbored only 3 genes, and pR16.0676_34k contained at least 12 genes related to conjugation. Fusion of the 2 plasmids caused the composite plasmid to become self-transmissible. When the composite plasmid was transferred back into a rifampin-resistant mutant of *Salmonella* Anatum strain R13.0957, we obtained transconjugants harboring only a 58-kb or 83-kb R plasmid, which were derived from the 125-kb plasmid through deletions (Appendix Figure, panel C). Accordingly, the composite plasmid was unstable in *Salmonella* Anatum.

## Conclusions

We identified a new *Salmonella* Anatum clone that emerged in Taiwan in 2011. During 2011–2014, strains of the new clone were not resistant and caused few infections. The dramatic increase in *Salmonella* Anatum infections that occurred during 2016–2017 was strongly associated with the emergence of MDR strains in 2015. The most crucial concern regarding emergence of the MDR *Salmonella* Anatum clone was that all MDR strains carry *bla*_DHA-1_, which encodes AmpC β-lactamase and confers resistance to β-lactam drugs, including third-generation cephalosporins. This resistance cannot be overcome by using β-lactam inhibitors. Because these MDR strains can cause numerous infections in humans and are prevalent in animals used for food, urgent control measures are needed.

Appendix Additional methods and results from study of new multidrug-resistant *Salmonella enterica* serovar Anatum clone, Taiwan, 2017–2017.
