# Supplementary material for: New Multidrug-Resistant Salmonella enterica Serovar Anatum Clone, Taiwan, 2015–2017
Source: Emerg Infect Dis. 2019 Jan;25(1):144–7. doi: 10.3201/eid2501.181103 (PMC6302611; doi:10.3201/eid2501.181103)
Supplement: Appendix — Additional methods and results from study of new multidrug-resistant Salmonella enterica serovar Anatum clone, Taiwan, 2017–2017. [file 18-1103-Techapp-s1.pdf]

Article DOI: https://doi.org/10.3201/eid2501.181103

New Multidrug-Resistant *Salmonella enterica* Serovar Anatum Clone, Taiwan, 2015–2017

Appendix

Experimental Methods

Antimicrobial susceptibility testing

We performed antimicrobial susceptibility testing for Salmonella isolates using the microbroth dilution method and custom-made Sensititre® 96 well susceptibility plates (TREK Diagnostic Systems Ltd., West Sussex, UK). The antimicrobials for the custom-made Sensititre plates changed several times during 2004—2016. The test was performed according to the manufacturer's instructions, and the interpretation of MIC results was followed the guidelines of the Clinical and Laboratory Standards Institute (CLSI) (*1*). The CLSI interpretive criteria were used for all of the antimicrobials except streptomycin, for which MIC≧32 μg/ml was used for streptomycin resistance.

Whole genome sequencing and sequence analysis

We conducted whole genome sequencing of *S.* Anatum isolates using Illumina MiSeq sequencing platform (Illumina Inc. USA) with MiSeq Reagent Kit v3 (2X 300 bp). Appendix Table lists the sequencing data (coverage and N50) and the NCBI accession numbers for the isolates with WGS data. We used the CLC Genomics Workbench software (Qiagen Bioinformatics, Germany) to assemble the Illumina reads for all isolates, identified resistance genes and incompatibility groups of plasmids using the ResFinder and PlasmidFinder tools provided by the Center for Genomic Epidemiology (http://www.genomicepidemiology.org/), and determined sequence type using the plugin tool provided in BioNumerics version 7.6.3 (Applied Maths Inc.).

Sequencing of complete genome of *S.* Anatum strain R16.0676 and plasmids

We used a MinION nanopore sequencer (Oxford Nanopore Technologies, UK) to obtain long reads for *S.* Anatum strain R16.0676 and plasmids from transconjugants, an Albacore basecaller (Oxford Nanopore Technologies) to execute base calling of nanopore reads, Canu (*2*) to assemble reads, Pilon (*3*) to polish the Canu-assembled contigs with the Illumina reads, and Nanopolish (https://github.com/jts/nanopolish) to polish the Canu-assembled contigs with raw nanopore reads. Subsequently, we used PCR and Sanger sequencing techniques to correct the uncertain sequences and RAST (http://rast.nmpdr.org/) to annotate the complete chromosome and plasmid sequences of the strain R16.0676 (*4*).

Construction of a dendrogram for *S.* Anatum strains using wgSNP profiles

We used the tools provided in BioNumerics version 7.6.3 for construction of a dendrogram with wgSNP profiles of *S.* Anatum strains. The sequences of raw reads were mapped to the reference genomic sequence of *S.* Anatum strain GT-38 (GenBank accession no. CP013226) and the mapped sequences of strains and the reference were aligned for SNP calling by using the option of strict SNP filtering (closed SNP set). By using this SNP calling criteria, SNPs are called by removing positions with at least one ambiguous base (non-ATGC base), one unreliable base (N), one gap and non-informative SNPs. Each retained SNP position has minimum 5x coverage, at least covered once in both forward and reverse direction. The minimum distance between retained SNP position is 12 bp. A dendrogram was constructed with the whole genome SNP profiles using the categorical (SNPs) option for similarity coefficient and single linkage algorithm for cluster analysis.

Conjugation

We conducted conjugation experiments to transfer the resistance genes-carrying (R) plasmid from strain R16.0676 into *Escherichia coli* C600 recipients by using LB medium with 50 mg/L ampicillin and 2,000 mg/L streptomycin for transconjugant selection. Subsequently, we transferred an R plasmid from an *E. coli* transconjugant back to a rifampicin-resistant mutant of *S.* Anatum strain R13.0957 by using LB medium with 50 mg/L ampicillin and 150 mg/L rifampicin for transconjugant selection. The

plasmids and their sizes were estimated using a S1-PFGE method (*5*). The sequences of R plasmids from transconjugants were determined using MinION nanopore sequencer or/and Illumina MiSeq sequencer.

Appendix References

1. Clinical and Laboratory Standards Institute (CLSI). Performance standards for antimicrobial susceptibility testing. 27th ed. Wayne (PA): Clinical and Laboratory Standards Institute; 2017.

2. Koren S, Walenz BP, Berlin K, Miller JR, Bergman NH, Phillippy AM. Canu: scalable and accurate long-read assembly via adaptive *k*-mer weighting and repeat separation. Genome Res. 2017;27:722–36. PubMed http://dx.doi.org/10.1101/gr.215087.116

3. Walker BJ, Abeel T, Shea T, Priest M, Abouelliel A, Sakthikumar S, et al. Pilon: an integrated tool for comprehensive microbial variant detection and genome assembly improvement. PLoS One. 2014;9:e112963. PubMed http://dx.doi.org/10.1371/journal.pone.0112963

4. Aziz RK, Bartels D, Best AA, DeJongh M, Disz T, Edwards RA, et al. The RAST Server: rapid annotations using subsystems technology. BMC Genomics. 2008;9:75. PubMed http://dx.doi.org/10.1186/1471-2164-9-75

5. Barton BM, Harding GP, Zuccarelli AJ. A general method for detecting and sizing large plasmids. Anal Biochem. 1995;226:235–40. PubMed http://dx.doi.org/10.1006/abio.1995.1220

Appendix Table 1. The NCBI accession numbers for the whole genome sequences of *Salmonella enterica* serova Anatum isolates and plasmids investigated in this study*

Strain/Plasmid	BioProject	BioSample	SRA	Coverage (X)	N50 (bp)
CA08.145	PRJNA478278	SAMN09788957	SRR7665411	29.4	332,460
CC04.028	PRJNA478278	SAMN09788958	SRR7665410	47.4	733,275
CC06.031	PRJNA478278	SAMN09788959	SRR7665409	33	432,006
CF09.078	PRJNA478278	SAMN09788960	SRR7665408	39.1	640,112
CH05.023	PRJNA478278	SAMN09788961	SRR7665415	44.3	695,804
CH07.062	PRJNA478278	SAMN09788962	SRR7665414	50.5	741,255
CI07.001	PRJNA478278	SAMN09788963	SRR7665413	73.3	741,487
CS182	PRJNA478278	SAMN09788964	SRR7665412	29.9	374,704
D013	PRJNA478278	SAMN09788965	SRR7665406	44.7	699,584
D020	PRJNA478278	SAMN09788966	SRR7665405	41	643,641
EA04.039	PRJNA478278	SAMN09788967	SRR7665354	42.8	678,360
EA04.047	PRJNA478278	SAMN09788968	SRR7665353	49.1	733,283
MS32850	PRJNA478278	SAMN09788969	SRR7665356	33.3	434,923
MS32915	PRJNA478278	SAMN09788970	SRR7665355	38.2	551,024
NC04.178	PRJNA478278	SAMN09788971	SRR7665358	45.9	732,987

Strain/Plasmid	BioProject	BioSample	SRA	Coverage (X)	N50 (bp)
NJ08.181	PRJNA478278	SAMN09788972	SRR7665357	35.9	531,861
NK04.008	PRJNA478278	SAMN09788973	SRR7665360	36.3	532,082
NL05.024	PRJNA478278	SAMN09788974	SRR7665359	44.2	695,569
P049	PRJNA478278	SAMN09788975	SRR7665351	54.1	741,359
P164	PRJNA478278	SAMN09788976	SRR7665350	31	399,915
P165	PRJNA478278	SAMN09788977	SRR7665385	24.6	173,037
PS23	PRJNA478278	SAMN09788978	SRR7665386	28.9	319,346
R13.0957	PRJNA478278	SAMN09788979	SRR7665387	40.3	643,625
R13.1215	PRJNA478278	SAMN09788980	SRR7665388	35.7	495,112
R13.1671	PRJNA478278	SAMN09788981	SRR7665381	29.3	332,460
R13.2266	PRJNA478278	SAMN09788982	SRR7665382	83.7	741,735
R14.1408	PRJNA478278	SAMN09788983	SRR7665383	44.2	695,795
R15.0600	PRJNA478278	SAMN09788984	SRR7665384	54.5	741,397
R15.0695	PRJNA478278	SAMN09788985	SRR7665378	56.5	741,426
R15.0913	PRJNA478278	SAMN09788986	SRR7665379	37.6	533,333
R15.1294	PRJNA478278	SAMN09788987	SRR7665364	35.7	495,179
R15.1365	PRJNA478278	SAMN09788988	SRR7665363	37.3	533,154
R15.1977	PRJNA478278	SAMN09788989	SRR7665362	31.4	405,893
R15.2697	PRJNA478278	SAMN09788990	SRR7665361	27.9	289,505
R16.0274	PRJNA478278	SAMN09788991	SRR7665368	30.9	399,664
R16.0348	PRJNA478278	SAMN09788992	SRR7665367	30.8	399,652
R16.0460	PRJNA478278	SAMN09788993	SRR7665366	43.3	694,259
R16.0569	PRJNA478278	SAMN09788994	SRR7665365	36.4	532,231
R16.0696	PRJNA478278	SAMN09788995	SRR7665370	34.5	454,735
R16.1070	PRJNA478278	SAMN09788996	SRR7665369	66.1	741,485
R16.1231	PRJNA478278	SAMN09788997	SRR7665395	33.6	452,715
R16.1486	PRJNA478278	SAMN09788998	SRR7665396	37.6	533,690
R16.2802	PRJNA478278	SAMN09788999	SRR7665393	30.1	387,305
R16.2821	PRJNA478278	SAMN09789000	SRR7665394	30	383,318
R16.2885	PRJNA478278	SAMN09789001	SRR7665391	38.6	639,698
R16.3115	PRJNA478278	SAMN09789002	SRR7665392	54.1	741,397
R16.3355	PRJNA478278	SAMN09789003	SRR7665389	56.3	741,426
R16.3623	PRJNA478278	SAMN09789004	SRR7665390	36.6	532,391
R16.3927	PRJNA478278	SAMN09789005	SRR7665403	33.5	437,722
R16.4304	PRJNA478278	SAMN09789006	SRR7665404	31.9	406,851
R16.4391	PRJNA478278	SAMN09789007	SRR7665376	38.3	551,278
R16.4880	PRJNA478278	SAMN09789008	SRR7665372	51	741,359
R17.0132	PRJNA478278	SAMN09789009	SRR7665349	41.6	643,695
R17.3086	PRJNA478278	SAMN09789010	SRR7665397	80.2	741,599
R17.3110	PRJNA478278	SAMN09789011	SRR7665375	33.6	454,461
R17.3140	PRJNA478278	SAMN09789012	SRR7665373	101	742,067
R17.3154	PRJNA478278	SAMN09789013	SRR7665380	79.4	741,597
R17.3160	PRJNA478278	SAMN09789014	SRR7665377	83.4	741,599
R17.3161	PRJNA478278	SAMN09789015	SRR7665352	81.1	741,599
R17.3203	PRJNA478278	SAMN09789016	SRR7665371	107.6	782,067
R17.3211	PRJNA478278	SAMN09789017	SRR7665407	95.4	741,778
R17.4426	PRJNA478278	SAMN09789018	SRR7665398	30.2	399,562
R17.4643	PRJNA478278	SAMN09789019	SRR7665399	50	733,496
R17.5171	PRJNA478278	SAMN09789020	SRR7665400	45.5	719,281

Strain/Plasmid	BioProject	BioSample	SRA	Coverage (X)	N50 (bp)
SA11.164	PRJNA478278	SAMN09789021	SRR7665401	32.7	427,327
SG06.139	PRJNA478278	SAMN09789022	SRR7665402	46.5	733,111
SN08.005	PRJNA478278	SAMN09789023	SRR7665374	25.8	209,293
R16.0676	PRJNA474787	SAMN09373897	SRR7665547	35.2	465,526
R18.1457	PRJNA478278	SAMN09914824	SRR7755901	82.7	741,599
R18.1458	PRJNA478278	SAMN09914823	SRR7755902	41.6	643,774

*R16.0676, GenBank accession no. CP029800; pR16.0676_34k, GenBank accession no. CP029801; pR16.0676_90k, GenBank accession no. CP029802; pConj125k, GenBank accession no. MK033499; pConj58k, GenBank accession no. MK033500; pConj83k, GenBank accession no. MK033501.

Appendix Table 2. Distribution of PFGE types and clonal lineages for *Salmonella enterica* serovar Anatum isolates collected during 2004–2017*

Lineage, sublineage, PFGE type	Distribution of isolates, by year														
	2004	2005	2006	2007	2008	2009	2010	2011	2012	2013	2014	2015	2016	2017	Total
L1															
SMX.082	9	4	4	6	2	2			1	1		1			30
SMX.087	6	3	2	4	3	2					2	2	3		27
SMX.097		2		2										1	5
SMX.092	1				1									1	3
Other 21 types	11	2	2	3	4					1				1	24
Subtotal	27	11	8	15	10	4	0	0	1	2	2	3	3	3	89
L3															
SL3_1															
SMX.768								1	1	3	3	4	33	26	71
SMX.871												6	8	2	16
SL3_2															
SMX.642										2		22	168	544	736
SMX.903												1	18	43	62
SMX.1052													2	17	19
SL3_1 and SL3_2															
Other 54 types										1	1	3	21	100	126
Subtotal	0	0	0	0	0	0	0	1	1	6	4	36	250	732	1,030
L2															
SMX.098		2													2
Total *S.* Anatum	27	13	8	15	10	4	0	1	2	8	6	39	253	735	1,121
All *Salmonella* collected	2,535	2,326	2,071	3,766	2,284	1,923	1,621	742	863	2,247	1,821	3,042	3,755	5,164	34,160

*L, lineage; PFGE, pulsed-field gel electrophoresis; SL, sublineage.

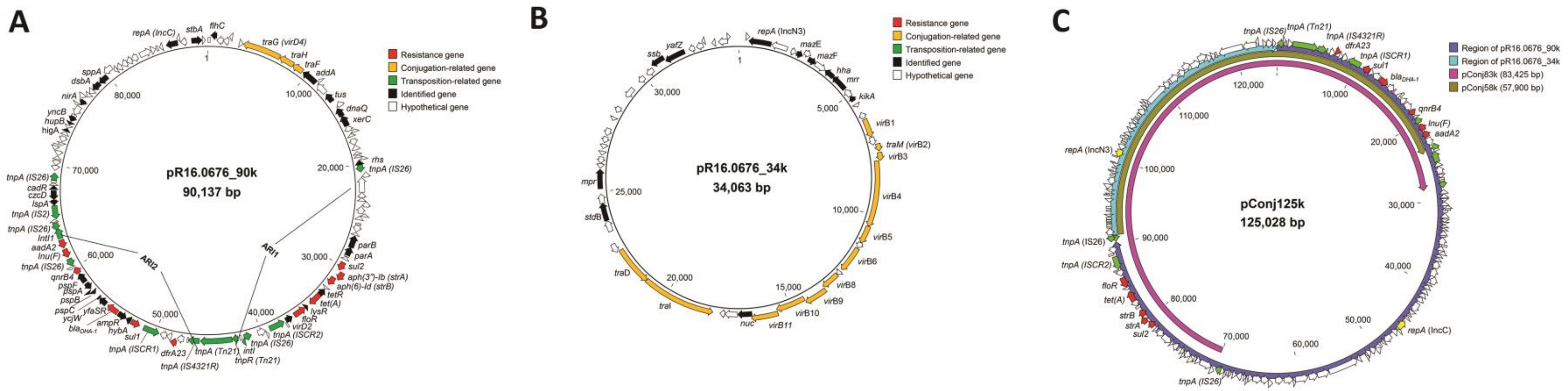

Appendix Figure. Genetic maps of plasmids pR16.0676-90k (A) and pR16.0676-34k (B) in *Salmonella enterica* serovar Anatum strain R16.0676 and pConj125k, pConj83k and pConj58k (C) from transconjugants.
